# Pemphigus foliaceus presenting with violaceous, painful nodules and an aberrant auto-antibody expression in a Latino patient

**DOI:** 10.1016/j.jdcr.2021.02.020

**Published:** 2021-03-02

**Authors:** Jeffrey N. Li, Parth Patel, Cristian Gonzalez, Travis Vandergriff, Heather Goff

**Affiliations:** Department of Dermatology, University of Texas Southwestern Medical Center, Dallas, Texas

**Keywords:** desmoglein, Latino, pemphigus foliaceus, pemphigus vulgaris, skin of color, DSG-1, desmoglein-1, DSG-3, desmoglein-3, PF, pemphigus foliaceus, PV, pemphigus vulgaris

## Introduction

Pemphigus foliaceus (PF) is an autoimmune blistering disorder characterized by scaly, crusted, cutaneous erosions without mucous membrane involvement, typically in a seborrheic distribution.^1^ Rarely, PF can present with generalization and even exfoliative erythroderma. Lesions originate from disruption of desmoglein-1 (DSG-1) by auto-antibody production, resulting in acantholysis in the stratum granulosum. PF is the second most common form of pemphigus disease behind pemphigus vulgaris (PV), which is characterized by mucous membrane involvement and auto-antibody production against desmoglein-3 (DSG-3) and sometimes DSG-1.^1^^,^^2^ PF is often initially misdiagnosed as various common dermatologic conditions, which can lead to a delay in correct diagnosis of months to even years.^3^ In this case report, we discuss a Latino patient with a histology consistent with PF whose serum was suggestive of PV with no evidence of typical PV mucosal or cutaneous manifestations.

## Case report

**Fig 1:**
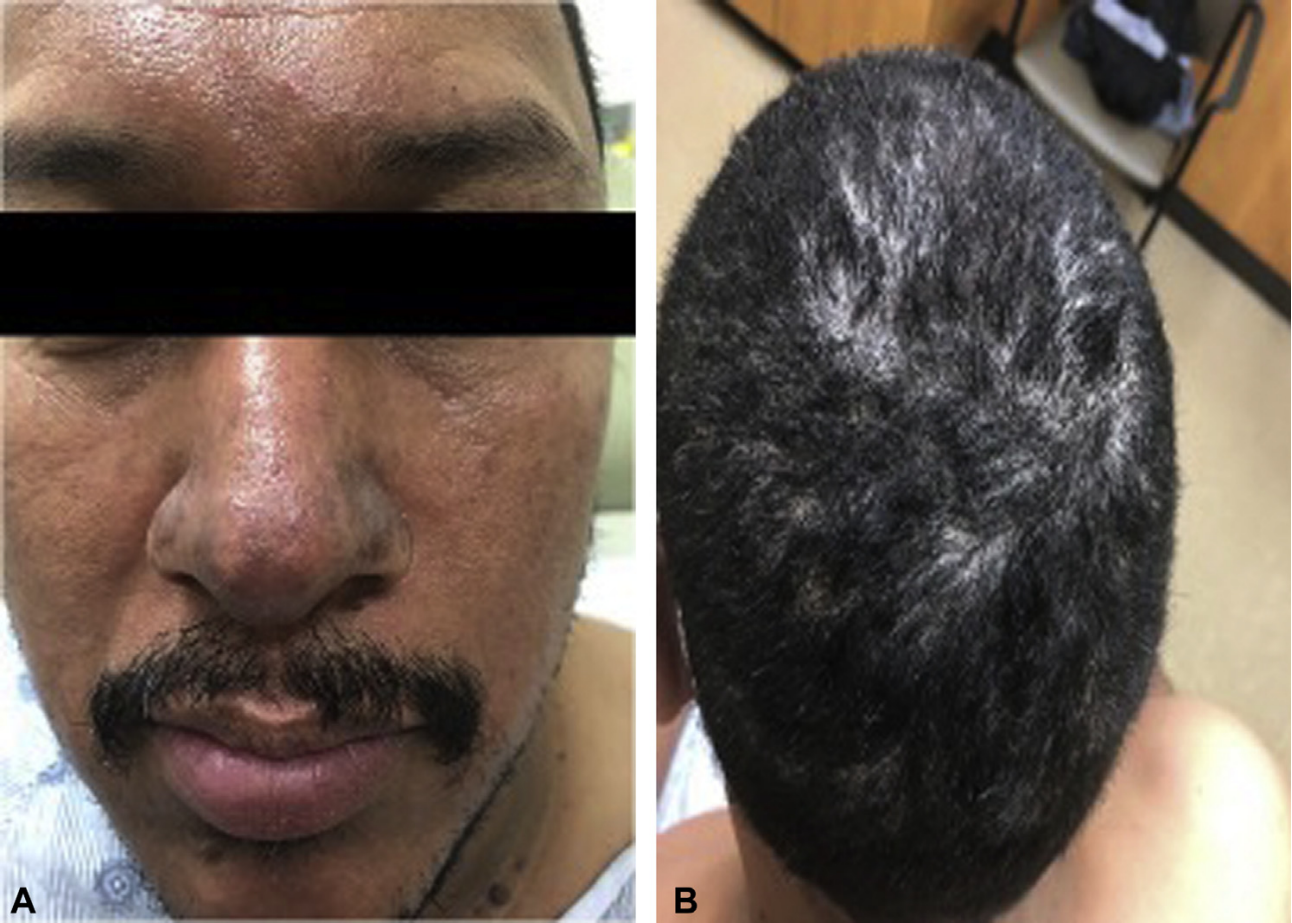
Photos from the initial clinic visit demonstrating violaceous nodules on the face and nonscarring scalp alopecia.

A 41-year-old Latino man with no prior medical history presented with a 1-year history of exquisitely painful violaceous nodules on the face and ears, in addition to nonscarring scalp alopecia (Fig 1). He denied any oral or genital lesions. Of note, he had previously presented to urgent care, where he was diagnosed with scalp folliculitis and treated with topical and oral antibiotics for 3 months. Differential diagnosis included bullous impetigo, dissecting cellulitis, PF, sarcoidosis, and Kaposi's sarcoma.

**Fig 2:**
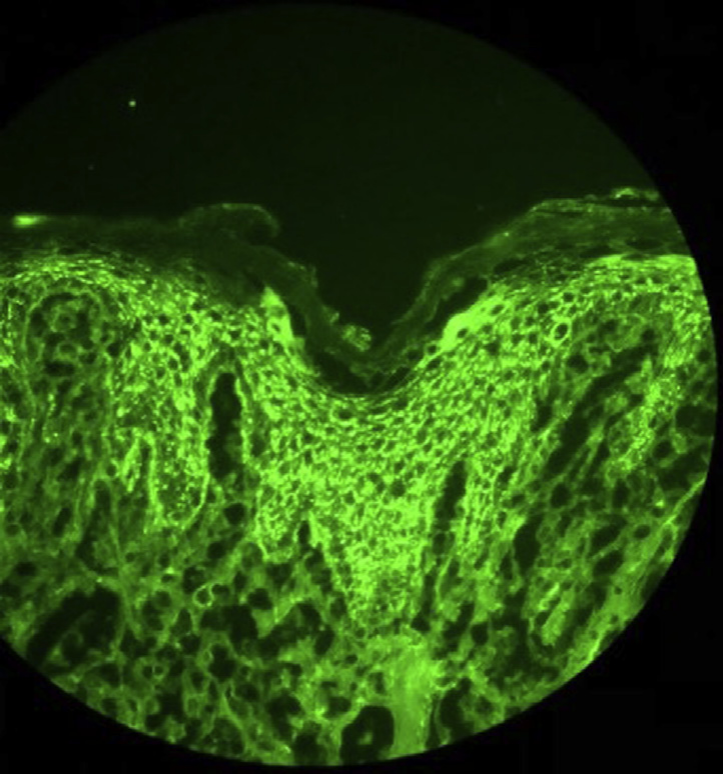
DIF revealing intercellular deposition of IgG and complement component 3 within the epidermis, consistent with a diagnosis of PF. *DIF*, Direct Immunofluorescence; *PF*, pemphigus foliaceus.

At his dermatology appointment, a biopsy was obtained from a ear nodule showing acanthosis with acantholysis of the granular cell layer, with direct immunofluorescence revealing intercellular deposition of IgG and complement component 3 within the epidermis, consistent with PF (Fig 2). Serology testing revealed not only a profound elevation of DSG-1 autoantibodies (174; normal, <20), but also an aberrant expression of DSG-3 autoantibodies (28; normal, <28). In addition, a test for antinculear antibodies was positive, speckled at 1:160.

**Fig 3:**
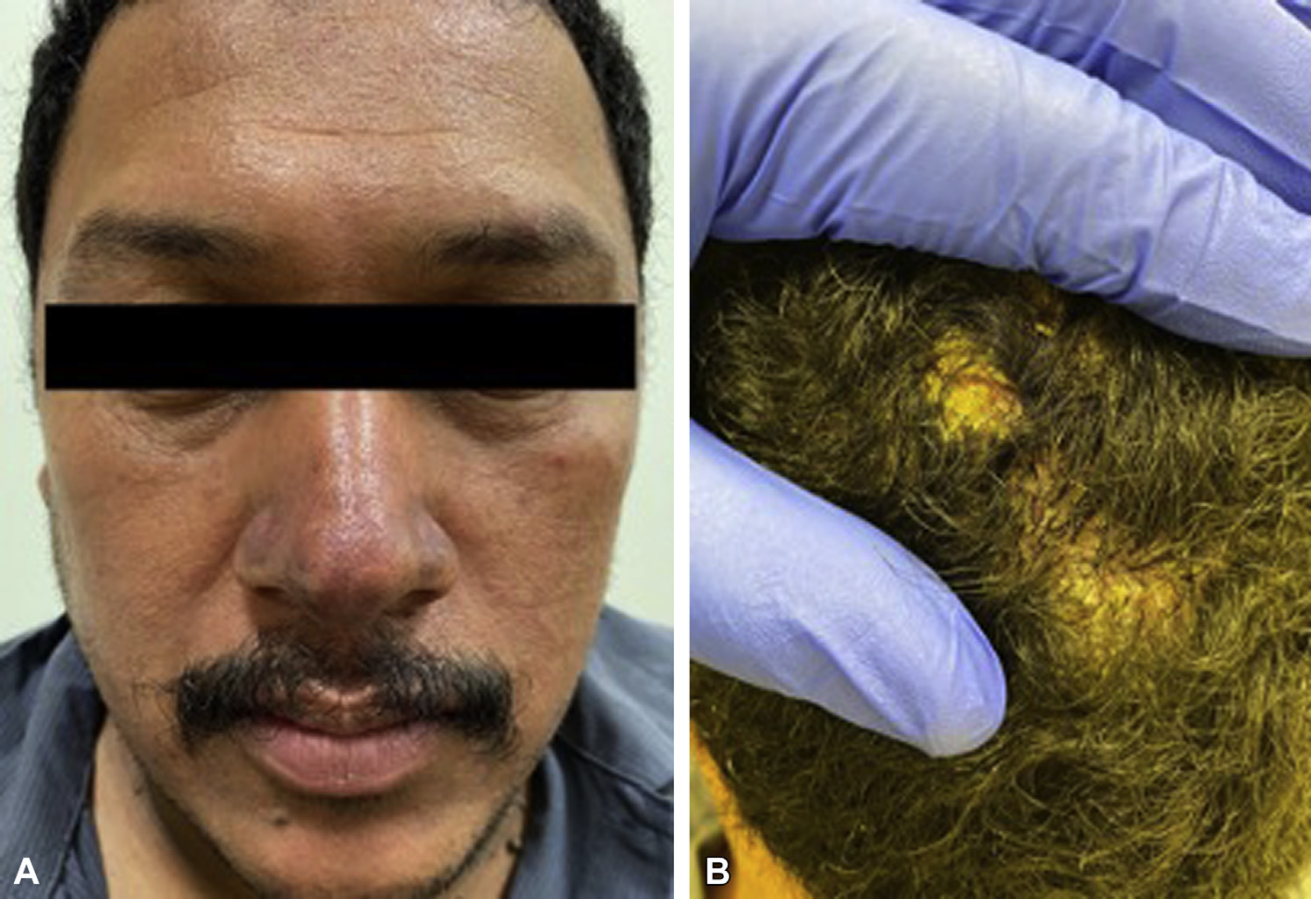
Photos from the 1-month follow-up visit with persistence of violaceous nodules and plaques with worsening scalp erosions.

The patient was started on 40 mg of prednisone daily with plans to start rituximab. At his 1-month follow-up, he was noted to have more significant painful erosions on the scalp and persistence of painful nodules and violaceous thin plaques on his ears and face, respectively (Fig 3). Bacterial swabs revealed bacterial superinfection with methicillin-sensitive *Staphylococcus aureus* and *Klebsiella* species. He was started on mycophenolate given reluctance to proceed with rituximab due to fear of increased susceptibility to COVID-19 and trimethoprim-sulfamethoxazole (800-160 mg once daily for 2 weeks) for superinfection with reduction of his prednisone dose with plans for close monitoring of clinical symptoms and follow-up.

## Discussion

Diagnosing PF is frequently a diagnostic challenge and often misdiagnosed as bullous impetigo, dissecting cellulitis of the scalp, inflammatory seborrheic dermatitis, among other common dermatologic conditions.^3^ In this case, the patient initially endorsed draining scalp pustules, resulting in a diagnosis of folliculitis. However, despite appropriate treatment for this condition, he continued to develop new scalp and face lesions, highlighting that PF may mimic other common dermatologic conditions, in particular when the scalp is involved.

Additionally, bacterial swabs performed at his follow-up visit revealed bacterial superinfection with methicillin-sensitive *Staphylococcus aureus* and *Klebsiella*, showing that PF may also occur concomitantly with other common dermatologic conditions. Though no data are available on the prevalence of superinfection in PF specifically, a study by Aghmiyuni et al^4^ found that 59.1% of PV patients had co-existing *S. aureus* infection, followed by *Staphylococcus epidermidis* at 28.1%. Diagnostic workup should include a thorough medical history (including medications), comprehensive mucosal and cutaneous exams, lesional biopsy for hematoxylin-eosin–staining, perilesional biopsy for direct immunofluorescence, serum collection for enzyme-linked immunosorbent assay, and bacterial and fungal cultures due to the high prevalence of superinfection.

Because of facial involvement, pemphigus erythematosus (PE) was also considered. PE, or Senear-Usher syndrome, is a controversial disease process with variable diagnostic criteria. It can be considered a subtype of PF and has been described as a localized or early-stage variant with mixed features of systemic lupus erythematosus and PF that can involve the malar regions. Serologic evaluation may demonstrate antinuclear antibodies titers, as in this case. However, histologic discordance was not consistent with PE. Immunoreactants were not deposited in the expected granular deposition, and keratinocyte nuclear fluorescence was not seen.^5^

Another layer of complexity in diagnosis was skin of color. Few case reports discuss the cutaneous presentation of PF in patients with skin of color.^3^ Due to the violaceous and infiltrative appearance of the patient's lesions, Kaposi sarcoma and sarcoidosis were considered, which could potentially delay diagnosis and result in unnecessary and expensive additional testing. It is important to recognize that erythema in darker-pigmented individuals can appear violaceous and that epidermal death can appear dusky or gray.^6^^,^^7^

Another layer of difficulty was management of PF during the COVID-19 pandemic. Though rituximab was offered, the patient had concerns beginning immunosuppression due to the unclear safety of rituximab affecting susceptibility to the viral infection. It is also uncertain whether disease-modifying therapy would hamper the effectiveness of any future COVID-19 vaccines and blunt antibody production.^8^

Additionally, serologies showed not only a profound elevation of DSG-1 autoantibodies, but also an aberrant expression of DSG-3 autoantibodies, without mucosal or cutaneous manifestations of PV. Though it is more commonly reported for PV patients to develop PF, there have been case reports of patients with an established diagnosis of PF developing PV months or several years later.^2^^,^^9^^,^^10^ This is theoretically due to epitope spreading, where in the initial stage, solely anti-DSG1 autoantibodies are present.^9^^,^^10^ During the disease course, DSG3 may be released from injured tissue, leading to recognition and production of anti-DSG3 antibodies. It is plausible that this patient may have been caught in this transition period from PF to PV. Patients with PF are usually treated less aggressively than PV. Given this patient's clinical and histopathologic discordance with serological findings and lack of response to systemic prednisone, we suggest this patient and those with similar presentations be monitored closely and treated more aggressively.

## Conclusion

PF is a rare and difficult clinical diagnosis to make, increasingly obscured in patients with skin of color. Awareness of differences in presentation is necessary to ensure expedient diagnosis and avoid delays in treatment. In addition, increased awareness of transition of PF to PV and *vice versa* can assist in approaching treatment decisions.

## Conflicts of interest

None disclosed.
